# Neither a Nitric Oxide Donor Nor Potassium Channel Blockage Inhibit RBC Mechanical Damage Induced by a Roller Pump

**DOI:** 10.2174/1874120700802010017

**Published:** 2008-04-01

**Authors:** Pinar Ulker, Herbert J Meiselman, Oguz K Baskurt

**Affiliations:** a Department of Physiology, Akdeniz University Faculty of Medicine, Antalya, Turkey; b Department of Physiology and Biophysics, Keck School of Medicine, University of Southern California, Los Angeles, CA, USA

## Abstract

Red blood cells (RBC) are exposed to various levels of shear stresses when they are exposed to artificial flow environments, such as extracorporeal flow circuits and hemodialysis equipment. This mechanical trauma affects RBC and the resulting effect is determined by the magnitude of shear forces and exposure time. It has been previously demonstrated that nitric oxide (NO) donors and potassium channel blockers could prevent the sub-hemolytic damage to RBC, when they are exposed to 120 Pa shear stress in a Couette shearing system. This study aimed at testing the effectiveness of NO donor sodium nitroprussid (SNP, 10^-4^ M) and non-specific potassium channel blocker tetraethylammonium (TEA, 10^-7^ M) in preventing the mechanical damage to RBC in a simple flow system including a roller pump and a glass capillary of 0.12 cm diameter. RBC suspensions were pumped through the capillary by the roller pump at a flow rate that maintains 200 mmHg hydrostatic pressure at the entrance of the capillary. An aliquot of 10 ml of RBC suspension of 0.4 L/L hematocrit was re-circulated through the capillary for 30 minutes. Plasma hemoglobin concentrations were found to be significantly increased (~7 folds compared to control aliquot which was not pumped through the system) and neither SNP nor TEA prevented this hemolysis. Alternatively, RBC deformability assessed by laser diffraction ektacytometry was not altered after 30 min of pumping and both SNP and TEA had no effect on this parameter. The results of this study indicated that, in contrast with the findings in RBC exposed to a well-defined magnitude of shear stress in a Couette shearing system, the mechanical damage induced by a roller pump could not be prevented by NO donor or potassium channel blocker.

## INTRODUCTION

Cellular components of blood can be exposed to high shear stresses when they are subjected to flow in artificial environments such as hemodialysis equipment, cardiopulmonary bypass circuits and artificial organs [1,2], and mechanical trauma may result from stress levels, at least in some areas of these artificial flow environments [1-4]. Red blood cells (RBC) are affected by this mechanical trauma, with the damage ranging from slight changes in ion transport through the cell membrane to total destruction of RBC (i.e., hemolysis). In general, shear stresses higher than 300 Pa result in hemolysis [5], while lower level of shear stress may induce structural and functional alterations in RBC including mechanical impairment [1,6,7].

The improved design of medical equipment utilizing extracorporeal flow circuits has helped to limit mechanical trauma to blood and hence has significantly reduced the extent of hemolysis [8]. However, even slight degrees of hemolysis may result in clinical problems such as adverse effects due to the nitric oxide (NO) scavenging effect of free hemoglobin [9]. Arginase relased from hemolyzed RBC may also contribute to disturbed NO metabolism [10]. Decreased NO availability not only influence vascular tonus, but also may affect RBC rheological properties [11] and platelet agglutination and coagulation mechanisms [12]. Therefore, any measures that may result in reductions of mechanical damage to blood would be expected to contribute to the development of improved medical procedures and devices that involve artificial flow environments.

It has been previously demonstrated that sub-hemolytic mechanical damage to RBC could be prevented, in part, by including NO donors or a non-specific potassium channel blocker in RBC suspensions exposed to 120 Pa shear stress [6]. The present study was designed to extend these studies in order to test the effectiveness of such agents for preventing mechanical damage in an artificial flow circuit that included a roller pump. Sodium nitroprusside (SNP) as an NO donor and the non-specific potassium channel blocker tetraethyl-ammonium (TEA) were used at concentrations that have been previously demonstrated to be most effective for preventing sub-hemolytic mechanical damage [6]**.**

## MATERIALS AND METHODS

### Blood Samples and Preparation

Venous blood samples were obtained from 10 healthy, human volunteers of both sexes, aged between 28-54 years, and anticoagulated with ethylnediaminetetra-aceticacid (EDTA; 1.5 mg/ml). The hematocrit of each sample was measured using the microhematocrit method (12,000 g, 5 min) and was adjusted to 0.4 l/l by adding or removing an appropriate volume of autologous plasma; the samples were centrifuged at 900 g for 5 minutes at room temperature (20 ± 2°C), if plasma removal was necessary. Viscosity of RBC suspensions was 3.9 ± 0.4 cP, measured at 750 sec^-1^ shear rate, using a Wells-Brookfield cone-plate viscometer (DV II + Pro, Brookfield Engineering Labs, Middleboro, MA, USA). Each sample was then divided into four aliquots of 10 ml and treated as detailed below.

### Flow System

**Fig. (1):**
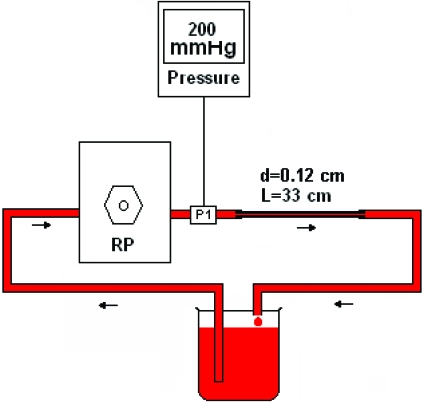
The flow system (RP: Roller pump; P1: Pressure transducer; See the text for details).

The flow system (Fig. **1**) included a roller pump (Masterflex Model 7521-01, Cole Parmer Instrument Co., Vernon Hills, IL, USA) and a glass capillary tube (diameter=0.12 cm, length=33 cm) with the roller pump connected to the capillary entrance through an in-line pressure transducer. The blood sample in the reservoir was recirculated through the capillary by the roller pump at a flow rate required to generate 200 mmHg pressure at the entrance of the capillary. The reservoir was covered with a plastic cap to prevent evaporation, and no drying of blood was observed during the experimental period. The temperature of the sample and capillary was maintained at 37 °C by immersing the reservoir and capillary into a water bath.

The wall shear stress in the capillary was calculated to be 24.2 Pa by using the following standard equation [13]:

$\tau =\frac{\Delta P\cdot D}{4\cdot L}$

where ΔP is the perfusion pressure (200 mmHg or 2.67 × 10^4^ Pa), d is the diameter of the capillary (0.12 cm) and L is the length of the capillary (33 cm). 

### Experimental Protocol

The four aliquots of 0.4 l/l hematocrit blood from each donor were treated as follows:

#### Control

This aliquot was kept at room temperature (20 ± 2°C) for one hour, and then transferred to the reservoir of the flow system described above, and the pump run for 30 minutes at 37 °C. The aliquot was removed from the system at the end of this period and used to determine plasma hemoglobin and RBC deformability;

#### SNP

Sodium nitroprussid (SNP, Sigma Chemical Co., item S0501) dissolved in phosphate buffered saline (PBS; pH 7.4) at a concentration of 10^-1^ M was added to this aliquot at a final concentration of 10^-4^ M, and then held at room temperature for one hour. The aliquot was run in the system for 30 minutes under conditions identical to Control then assayed for hemolysis and RBC deformability;

#### TEA

Tetraethlyammonium (TEA, Sigma T2265) dissolved in PBS at a concentration of 10^-4^ M was added to obtain a final concentration of 10^-7^ M then treated exactly like the SNP sample;

#### Control - No Flow

This aliquot was held for 60 minutes at room temperature and 30 minutes at 37°C, but was not subjected to flow as done for the Control sample.

### Determination of Plasma Hemoglobin

Aliquots of blood were centrifuged at 900 g for five minutes at room temperature (20 ± 2°C), and plasma was harvested. Two hundred μl of plasma was mixed with 800 μl of Drabkin’s solution (1.13 mM KH_2_PO_4, _0.6 mM K_3_[Fe(CN)_6, _0.8 mM KCN). Absorbance was measured at 540 nm and hemoglobin concentration was calculated using a calibration curve.

### Assesment of RBC Deformability

RBC deformability was determined at various fluid shear stresses by laser diffraction analysis using an ektacytometer (LORCA, RR Mechatronics, Hoorn, The Netherlands). The system has been described elsewhere in detail [14]. Briefly, a low hematocrit suspension of RBC in an isotonic viscous medium (4% polyvinylpyrrolidone 360 solution, MW= 360 kDa) is sheared in a Couette system composed of a glass cup and a precisely fitting bob, with a gap of 0.36 mm between the cylinders. A laser beam is directed through the sheared sample and the diffraction pattern produced by the deformed cells is analyzed by a desktop personal computer, which also controls the stepper motor that generates the pre-determined shear stresses. All measurements were done at 37 °C. Based upon the geometry of the elliptical diffraction pattern, an elongation index (EI) is calculated as: EI = (L-W)/(L+W), where L and W are the length and width of the diffraction pattern.

EI values, determined for nine shear stresses between 0.3 – 50 Pa, were used to calculate the shear stress required for half-maximal deformation (SS_1/2_) by applying a Lineweaver-Burk analysis procedure [15]. Impaired RBC deformability leads to increased SS_1/2 _values; SS_1/2 _values are used herein since the presentation and comparison of data *via *this approach are more convenient than *via *merely displaying shear stress-EI curves.

### Statistics

Values are expressed as mean ± standard error. One-way ANOVA followed by Dunnett post-test was used for comparisons between aliquots, using GraphPad Prism 4.0 Software.

## RESULTS

Plasma hemoglobin values for the four aliquots are presented in Fig. (**2**). The Control-No Flow aliquot level was 53.7 ± 3.6 mg/dl and increased to 364.4 ± 30.8 mg/dl in the aliquots pumped for 30 minutes (p<0.001). Inclusion of SNP or TEA at the concentrations given in the Materials and Methods section did not prevent this increment in plasma hemoglobin after 30 minutes of pumping. No statistically significant differences were found between the SNP or TEA and Control aliquots.

Fig. (**3**) demonstrates RBC deformability expressed as SS_1/2 _values. No significant differences in RBC deformability were detected among the aliquots after 30 minutes of pumping through the flow system, and hence no effects of SNP or TEA were evident. Note that pilot studies (not shown) indicated no significant differences from Control-No Flow for plasma hemoglobin values and SS_1/2 _values in non-pumped aliquots containing SNP or TEA.

## DISCUSSION

The present results demonstrate that the flow system used in this study induces significant RBC hemolysis, with a nearly seven-fold increase of plasma hemoglobin level after 30 minutes of pumping. Note that although preliminary experiments indicated that the level of hemolysis during pumping increased linearly with time (data not shown), no meaningful differences from the relations shown in Figs. (**2** and **3**) were detected: a 30 minute pumping period was selected for experimental efficiency. It should also be noted that the calculated wall shear stress as used herein was 24.2 Pa (i.e., 200 mmHg pressure gradient across the capillary) and thus markedly below the previously reported hemolytic threshold of 300 Pa [5].

The effects of RBC mechanical damage, as indicated by the significantly increased plasma hemoglobin concentration, could not be prevented by the NO donor SNP or the potassium channel blocker TEA. NO has been previously shown to play a significant role in the maintenance of normal RBC mechanical properties [11,16], and it has been proposed that this effect may be partly mediated by the potassium permeability of the RBC membrane [11]. Further, both NO donors and non-specific potassium channel blockage were previously found to be effective in preventing reduction of RBC deformability following exposure to sub-hemolytic shear stress [6]. However, several experimental conditions differed between that study [6] and the current work: 1) In the prior study RBC were exposed to 120 Pa [6] and thus to a five-fold higher level than used herein; 2) RBC were constantly subjected to shear for 120 sec in a Couette geometry system [6], whereas RBC were only exposed to the calculated shear stress intermittently while passing through the capillary; 3) The flow rate in the capillary tube can be calculated to be about 1 ml/sec using Poiseuille equation. Therefore, the exposure time of RBC to the calculated 24.2 Pa stress during each passage through the capillary was estimated to be about 0.35 sec with a total exposure time during the 30 minutes pumping time to be about of 67 sec, and hence 50% less when compared to the 120 sec constant shear in the Couette geometry.

Although the abovementioned experimental differences make exact comparisons to other work difficult, our use of a mechanical roller pump to generate flow represents a major departure from the prior experimental protocol [6]. It is not possible to exactly determine the magnitude of the mechanical forces due to the roller pump, but it could easily be shown that the roller pump itself was the main source of hemolytic mechanical trauma in the present flow system. Fig. (**4**) demonstrates the hemolytic effects of the flow system with and without the capillary: when operated with the capillary the inlet pressure was 200 mmHg, whereas with the pump operating at the same flow without the capillary the perfusion pressure was only a few mmHg. As can be seen in Fig. (**4**), the degree of hemolysis was only about 30% higher if the capillary was present in the flow circuit. Therefore, the majority of the mechanical damage in the current flow system is due to events occurring in or near the roller pump rather than to simple shear in the capillary, suggesting the possibility of different mechanisms for cell damage between the current study and brief steady shear in a Couette system [6].

**Fig. (2):**
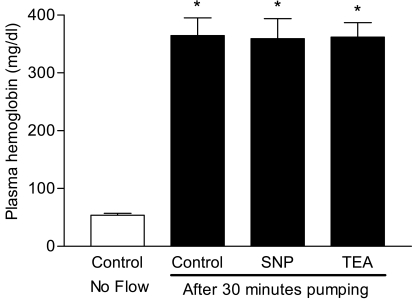
Plasma hemoglobin values in aliquots of blood containing sodiumnitroprusside (SNP, 10^-4^ M) and tetraethylammonium (TEA, 10^-7^ M) compared with control aliquots with or without pumping for 30 minutes. Values are presented as mean ± standard error (n=10). Values were significantly higher in all aliquots pumped for 30 minutes compared to the aliquot that was not pumped (*:p<0.001 compared to Control-No Flow).

**Fig. (3):**
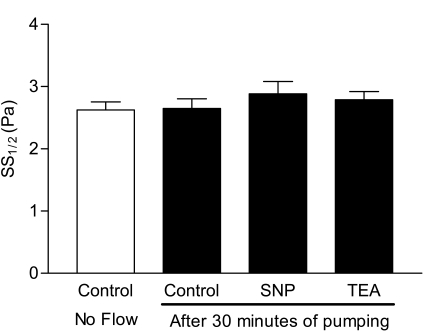
RBC deformability expressed as “shear stress at half maximal deformation” (SS_1/2_) in the aliquots of blood containing sodiumnitroprussid (SNP, 10^-4^M) and tetraethylammonium (TEA, 10^-7^M) compared with control aliquots with or without pumping for 30 minutes. Values are mean ± standard error (n=10).

Given the very large increase of plasma hemoglobin levels following 30 minutes of pumping (Fig. **2**), it is interesting to speculate as to why this level of hemolytic damage did not affect RBC deformability (Fig. **3**) whereas sub-hemolytic stress levels do increase cell rigidity [6]. One possible explanation relates to the type of mechanical damage in the two systems: 1) cell damage without hemolysis may be possible to detect using these cells in the LORCA laser diffraction system [14]; 2) mechanical damage resulting in hemolysis and cell fragmentation would not be detected since only intact RBC are sensed by the LORCA. That is, decreased deformability for mechanically impaired but intact RBC would be observed, whereas the measured deformability of normal, intact cells would not be affected by the presence of small cell fragments in the suspension. Note that this explanation “begs the question” regarding the mechanical behavior of non-hemolyzed RBC in the present study: by definition, these cells were exposed to sub-hemolytic stress levels, yet unlike our prior findings [6], have unaltered deformability (Fig. **3**). Possible answers to the “question” include partial hemolysis rather than an all or none mode of hemoglobin loss, or that only a small, non-detectable sub-population of intact RBC had reduced deformability. Of course, differences in the magnitude, detailed nature and duration of applied mechanical forces must also play a role.

In overview, our results indicate that: 1) neither the NO donor SNP nor the potassium channel blocker TEA are effective for preventing hemolytic mechanical trauma in a flow system that includes a roller pump; 2) the damage to RBC is determined by the stress magnitude, exposure time and flow details in the system and that knowledge of stress levels in all parts of a flow system must be considered; 3) additional studies are needed in order to fully define the mechanical factors that induce hemolysis and/or cell rigidity.

## Figures and Tables

**Fig. (4):**
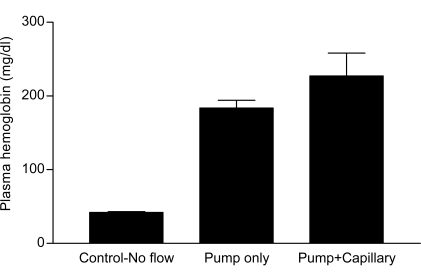
Plasma hemoglobin concentration in 0.4 l/l hematocrit blood following 30 minutes of flow with (“Pump+Capillary”) and without (“Pump only”) the capillary inserted into the flow system; the Control-No flow value represents no flow through the system. The flow rate was the same for both “Pump only” and “Pump+Capillary” experiments, but the pressure was close to zero if the capillary was absent and 200 mm Hg with the capillary inserted. Values are mean ± standard error. There was no significant difference between “pump only” and “pump+capillary” experiments.
